# Whole-genome sequencing and biocontrol functional analysis of *Pseudomonas chlororaphis* XZL-8 for controlling poplar canker pathogen *Cytospora chrysosperma*

**DOI:** 10.3389/fmicb.2026.1973011

**Published:** 2026-10-02

**Authors:** Huayi Huang, Yi Zhao, Chenglong Gao, Lili Hu, Danyang Zhao

**Affiliations:** Guangdong Academy of Forestry/Guangdong Provincial Key Laboratory of Forest Silviculture, Protection and Utilization/Key Laboratory of National Forestry and Grassland Administration on Ecosystem Conservation and Restoration in the Guangdong-Hong Kong-Macao Greater Bay Area, Guangzhou, China

**Keywords:** antifungal activity, biological control, poplar canker, *Pseudomonas chlororaphis* subsp. *aureofaciens*, secondary metabolites, volatiles, whole-genome analysis

## Abstract

**Introduction:**

Poplar canker triggered by *Cytospora chrysosperma* severely damages forest production, and screening efficient antagonistic bacteria is a sustainable strategy for eco-friendly disease management. This study aimed to characterize the taxonomic status, genomic features and biocontrol potential of strain XZL-8.

**Methods:**

Polyphasic identification and whole-genome sequencing were conducted to confirm the species of strain XZL-8. Functional annotation against NR, COG, GO and KEGG databases and antiSMASH secondary metabolite prediction were carried out to decode its functional genes. A series of *in vitro* antagonistic experiments, as well as detached branch inoculation assays, were implemented to verify its phenotypic traits.

**Results:**

Strain XZL-8 was classified as *Pseudomonas chlororaphis* subsp. *aureofaciens*. A total of 22 secondary metabolite biosynthetic gene clusters were predicted in its genome; the gene clusters encoding hydrogen cyanide and pyrrolnitrin synthesis shared high homology with known reference clusters, with similarity coefficients reaching 0.85 and 0.99, respectively. Phenotypic assays showed that the strain inhibited the pathogen via direct antagonism, extracellular metabolites, and volatile substances. Genomic analysis further uncovered candidate genes linked to plant-beneficial properties such as hydrolytic enzyme production, HCN synthesis, and phosphate solubilization. Detached-branch assays confirmed its promising protective effect against woody stem canker.

**Conclusion:**

Strain XZL-8 harbors abundant genetic determinants putatively linked to biocontrol and plant-beneficial traits. *In vitro* and detached-branch tests demonstrated its biocontrol potential against *C. chrysosperma*, and therefore it is a promising candidate for developing microbial agents for the management of woody stem canker.

## 1 Introduction

Poplars trees (*Populus* spp.) are fast-growing and high-yielding trees that are widely distributed in Asia, Europe, and North America (Böhlenius et al., 2016). China has the largest area of poplar plantations in the world. China over 53 native and cultivated poplar species, with poplar plantations exceeding 7 million hectares and occupying 73% of the global total poplar cultivation area (Yang et al., 1998). Poplar trees has an important role in farmland shelterbelts, soil conservation, and environmental protection (Boothroyd-Roberts et al., 2013; Zhong et al., 2025). Poplar canker (*Cytospora* canker of poplar), caused by *Cytospora chrysosperma* (Pers.) Fr. (teleomorph: V*alsa sordida* Nit.), has seriously affected the development of the worldwide poplar industry (Alonso et al., 2000; Wang and Wang, 2020). Poplar canker mainly occurs in Northern China, where mortality rates of poplar reach more than 70% when it occurs severely. From 2006 to 2013, Four large-scale poplar canker diseases occurred in Liaoning Province, resulting in a total disaster area of more than 87,000 hm^2^, a death area of nearly 27,000 hm^2^, and a direct economic loss of more than 2 billion yuan. Unfortunately, the occurrence of poplar canker in Northeast China is more and more aggravated and frequent (He et al., 2020; Lin et al., 2023).

Currently, using chemical fungicides is still the most effective method for controlling poplar canker in China. However, the excessive and long-term use of chemical fungicides that are resistant to degradation is environmentally unfavorable and can lead to the evolution of resistance in pathogenic fungi (Fernando et al., 2005; Islam et al., 2024). Therefore, many studies have focused on developing and applying alternative methods that are less reliant on fungicides and more environmentally friendly.

Compared to chemical control, biological control offers a more effective and environmentally-friendly alternative methods to control poplar canker by used naturally isolated beneficial microorganisms. A number of bacterial strains have been exploited for their biocontrol potentials, particularly the genera *Bacillus* (Compant et al., 2005; Shafi et al., 2017), *Pseudomonas* (Raio et al., 2011; Morohoshi et al., 2022; Di Rico et al., 2026), and *Streptomyces* (Olanrewaju and Babalola, 2019; Wang et al., 2026). The genus *Pseudomonas* is a group of Gram-negative bacteria widely distributed and functionally diverse in the plant rhizosphere, and also one of the most valuable microbial resources for the biological control of plant diseases (Alattas et al., 2024). With strong rhizosphere colonization ability, abundant secondary metabolites, and multiple biocontrol mechanisms, strains of this genus play crucial roles in suppressing plant pathogens, improving soil microecology, and promoting plant growth (Dobrzyński and Jakubowska, 2025). As a representative species of *Pseudomonas* with excellent biocontrol characteristics, *Pseudomonas chlororaphis* has become a core strain resource for the development of microbial inoculants and biopesticides due to its strong antibacterial activity, broad antimicrobial spectrum, environmental friendliness, and safety to plants (Raio and Puopolo, 2021). *P. chlororaphis* can directly inhibit the growth of pathogens by producing antibacterial metabolites including phenazines, lipopeptides, and siderophores, block pathogen infection through niche and nutrient competition, and simultaneously induce systemic resistance in plants and promote nutrient absorption, achieving the synergistic effect of “disease control + growth promotion” (Arrebola et al., 2019).

The objectives of this study were to screen new strains of bacteria from the rhizospheres of poplar trees. The biocontrol potential of newly isolated strain to against *C. chrysosperma* on poplar trees was evaluated by determining the suppression of the hyphal growth and disease development on detached twigs. After screening, we identified and characterized the strain showing the highest antifungal activity for antagonistic, enzymatic, and plant growth-promoting potential.

## 2 Materials and methods

### 2.1 Fungal phytopathogen

*C. chrysosperma* CFCC89600 was provided by and maintained at the China Forestry Culture Collection Center (CFCC). The fungus was maintained on potato dextrose agar (PDA) slants at 4 °C and grown on PDA plates at 28 °C when used.

### 2.2 Isolation and screening of antagonistic bacteria

Soil samples were collected from the rhizosphere of healthy poplar trees in Haidian district of Beijing city, China. A soil sample (approximately 5 g) was transferred to a 100 mL Erlenmeyer flask containing 45 mL of sterilized water and shaken at 200 rev min^−1^ for 20 min prior to use (Kurabachew and Wydra, 2013). Serial dilutions of the soil suspension were spread onto Luria-Bertani (LB) agar plates, and the plates were incubated at 30 °C in darkness. A single bacterial colony, well separated from the other colonies, was selected and re-streaked onto a new LB agar plate with an inoculating loop; the plate was incubated at 30 °C for 24 h, and then stored at −80 °C in LB with 30% glycerol (v/v).

The antagonistic ability of bacteria against *C. chrysosperma* was measured by the dual culture assay described by Ferreira et al. (1991). The inhibition distance was defined as the width of the inhibition zone between the bacterial and fungal strains. The rhizosphere bacterial strain showed the highest antifungal activity against *C. chrysosperma* was selected for further studies.

### 2.3 Genome sequencing, annotation and analysis

The genomic DNA of strain XZL-8 was initially extracted using Bacteria DNA Kit (OMEGA) according to the manufacturer’s instructions. Sequencing was performed by Qingke Biotech (Beijing, China). Genome sequencing of strain XZL-8 was performed using a hybrid strategy combining Illumina NovaSeq 6000 and PacBio platforms. Illumina paired-end libraries (400 bp insert) were constructed and sequenced (2 × 150 bp) for short-read data, while PacBio 20 kb insert libraries were prepared and sequenced on the PacBio RS system for long reads. The raw paired end reads were trimmed and filtered using Trimmomatic 0.36 with the parameters SLIDINGWINDOW:4:15 and MINLEN:75 to obtain high-quality clean data.^1^ Hybrid de-novo genome assembly was performed with the Unicycler pipeline (v0.4.8), using both Illumina short-read and Oxford Nanopore long-read datasets. Briefly, Unicycler first invoked SPAdes (v3.15.4) to generate an initial short-read-based assembly graph from quality-filtered Illumina reads. Nanopore long-reads were then incorporated to construct sequence bridges, which span repetitive genomic regions and resolve complex structures for complete genome reconstruction. The bridging step was internally implemented by Miniasm (v0.3-r179) and Racon (v1.4.3) within the Unicycler workflow. Finally, iterative base-level polishing was conducted using Pilon (v1.24) with high-quality Illumina short-reads to correct mismatches and small indels. The quality and completeness of the final assembly were assessed by QUAST (v5.2.0) and BUSCO (v5.4.5). The final complete genome was visualized with Circos.^2^

Gene models of strain XZL-8 were obtained via ab initio prediction using GeneMark. All predicted gene models were functionally annotated via blastp searches against the NCBI non-redundant protein database (NR), SwissProt,^3^ KEGG,^4^ and COG.^5^ Biosynthetic gene clusters for secondary metabolites were identified using antiSMASH v7.1.^6^ tRNAs and rRNAs were predicted by tRNAscan-SE v1.23^7^ and RNAmmer v1.2,^8^ respectively.

### 2.4 ANI and GGDC analyses

Average nucleotide identity (ANIb) and digital DNA–DNA hybridization (dDDH) were calculated to clarify the taxonomic position of strain XZL-8 at both species and subspecies levels against reference genomes of *Pseudomonas chlororaphis* subspecies. Species boundaries were defined by thresholds of 95–96% ANI and 70% dDDH. A dDDH threshold of 79% and ANI > 98% were used as reference cut-offs for subspecies delineation. Whole-genome sequences of the strains were downloaded from the GenBank database,^9^ and genome sequences are listed in Table 1. ANI analysis was performed using the online platform JspeciesWS.^10^ The dDDH values were computed via the Type (Strain) Genome Server (TYGS)^11^ using the recommended formula 2 for draft-quality genome assemblies. The heatmaps of the ANI and dDDH values were generated using TBtools v2.485 (Chen et al., 2023).

**Table 1:** The GenBank accession numbers of genome sequences for *P. chlororaphis* spp.

Strain	Accession numbers
*P. chlororaphis* subsp. *phenazini* S1Bt23	CP139026
*P. chlororaphis* subsp. *chlororaphis* DSM 50083	CP027712
*P. chlororaphis* subsp. *aureofaciens* DSM 6698	CP027720
*P. chlororaphis* subsp. *aurantiaca* DSM 19603	CP027746
*P. chlororaphis* subsp. *piscium* DSM 21509	CP027707
*P. protegens* CHA0	CP003190
*P. saponiphila* DSM 9751	FNTJ01000004

### 2.5 Identification of strain XZL-8

Preliminary identification of strain XZL-8 was performed through morphological characterization and a series of physiological and biochemical analyses. The detected phenotypic traits included colony morphology, Gram staining, oxidase, catalase and lecithinase activities, Voges-Proskauer and methyl red reactions, starch and casein hydrolysis, carbon source utilization, nitrate reduction and H₂S generation, etc. (Sneath, 1986; Dong and Cai, 2001; Furuya et al., 2011). Bacterial growth tolerance to temperature, pH, and salinity was determined in liquid LB medium with three biological replicates. For temperature tolerance, the strain was shake-cultured at 4 °C, 10 °C, 30 °C, and 41 °C under identical culture conditions. For pH tolerance assessment, bacterial growth was detected in LB media adjusted to pH 4.0, 5.7, and 10.0. For salinity tolerance, the strain was cultured in LB media supplemented with 0.2, 2, and 5% NaCl, respectively. After incubation, bacterial growth status was evaluated by observing medium turbidity and biomass accumulation.

Genomic data were submitted to Type Strain Genome Server (TyGS) to automatically generate 16S rRNA and whole-genome phylogenetic trees with default algorithms and parameters. A total of 100 pseudo-bootstraps were performed, and trees were midpoint-rooted. Tree visualization and annotation were performed using FigTree v1.4.4.

### 2.6 Antifungal activity of strain XZL-8

#### 2.6.1 Antifungal effects of fermentation broth and sterile filtrate

Strain XZL-8 was cultured in 250 mL Erlenmeyer flasks with 100 mL of KMB at 30 °C on a rotary shaker at 200 rev min^−1^ for 24 h. The bacterial suspension was adjusted to approximately 1.0 × 10^9^ colony forming units per milliliter (cfu mL^−1^) prior to use 1 mL of the bacterial suspension of strain XZL-8 were transferred to 100 mL of TYB in 500 mL Erlenmeyer flask and incubated on a rotary shaker at 200 rev min^−1^ at 30 °C for different days to produce fermentation broth. Then, the fermentation broth was centrifuged, and the supernatant was filtered through a sterile membrane filter (0.22 μm; Pall, Ann Arbor, USA) to obtain the sterile culture filtrate.

The effect of fermentation broth and sterile culture filtrate of strain XZL-8 on hyphal growth of *C. chrysosperma* were assayed by measuring the mycelial growth inhibition rates (Song et al., 2012). Briefly, 2 mL of fermentation broth or sterile culture filtrate collected at each cultivation time point (1, 2, 3, 4, 5, 6, and 7 days) was mixed with 20 mL of PDA at 40–45 °C in a culture plate; for the control, sterile water was added. After the medium was solidified, we inoculated mycelial plugs (6 mm), collected from freshly growing fungal culture of *C. chrysosperma* using a sterile steel borer, onto the center of the plate. The plates were incubated at 28 °C. The diameters of the fungal colonies were measured every day until the control colonies covered more than three-quarters of the diameter of the dish. The criss-cross method was used to measure colony diameter. The percentage of growth inhibition was calculated using the following formula (Yang et al., 2011):


P(%)=100×[(C−d)−(T−d)]/(C−d)

where P is the inhibitory rate, C is the diameter of a control colony, T is the diameter of a treated colony, and d is the diameter of the mycelial plug.

#### 2.6.2 Suppression of hyphal growth of *C. chrysosperma* by volatiles

The effect of the volatiles of strain XZL-8 on hyphal growth of *C. chrysosperma* was tested by co-cultivating the fungi and bacteria on two-compartment plates (Yuan et al., 2012). The bacterial cells of strain XZL-8 was mixed with KMB medium at 40–45 °C, and filled one-half of each plate, for the control, sterile water was added. After the medium was solidified, the other half of plate was filled with PDA. After 24 h later, a 6 mm diameter fungal plug of *C. chrysosperma* was placed on the PDA side, then the plates were sealed with parafilm and incubated at 30 °C. The experiment was repeated three times. The diameters of fungal colonies were measured every 12 h and the percentages of growth inhibition were calculated as described above. The radial mycelial growth of *C. chrysosperma* was recorded at 0 h, 12 h, 24 h, 36 h, and 48 h to quantify growth suppression induced by bacterial volatile metabolites.

Mycelial growth values of the CK group (blue area) and volatile treatment group (red area) at each time gradient were extracted and plotted on a pentagonal radar map. The radial axis represents pathogen mycelial growth magnitude; smaller area coverage indicates stronger antifungal activity of bacterial volatiles. Three biological replicates were included for statistical reliability.

### 2.7 Production of hydrolytic enzymes

Cellulase production of strain XZL-8 was tested according to Essghaier et al. (2009). The activities of β-1,3-glucanase, chitinase, protease, and lipase were determined using poria powder medium, 0.2% colloidal chitin plate, skim milk agar plate, and 1% Tween 80 agar plate, respectively (Sock et al., 1990; Dong and Cai, 2001; Kamensky et al., 2003). Briefly, a single bacterial colony collected from an overnight culture was seeded onto the center of the plates; the plates were monitored for the appearance of a clear zone or aureola around the developing bacterial colonies at 28 °C.

### 2.8 Production of hydrogen cyanide (HCN)

The production of hydrogen cyanide (HCN) was determined qualitatively (Oh et al., 2011). Tryptic soy agar (TSA) medium supplemented with 4.4 g L^−1^ glycine was used to determine the production of HCN. To prevent the volatilization of gaseous HCN, the plates were sealed with Parafilm and incubated at 28 °C for 3 days. The development of an orange-to-brown color indicated HCN production.

### 2.9 Phosphate solubilization capacity

Phosphate solubilization ability was evaluated on the standard National Botanical Research Institute’s Phosphate (NBRIP) growth agar medium (Nautiyal, 1999). After 14 days of incubation at 28 C, the presence of colonies with clear halos was used as an index of phosphate solubilization.

### 2.10 Biocontrol activity on detached twigs

To evaluate the biocontrol activity of strain XZL-8 toward poplar canker caused by *C. chrysosperma*, uniform one-year-old twigs collected from poplar (Populus × euramericana) were used. The twigs were cut into 20 cm segments in length, immersed in 75% alcohol for 10 min for surface sterilization, and then washed with sterile water three times. The top of the twig is sealed in hot liquid wax. *C. chrysosperma* inoculations were conducted by a scald-inoculation method (Zang et al., 2007). The twigs were scalded for a 6 mm in diameter round area at twig mid-points using a soldering iron. The fermentation broth (1.0 × 10^5^ cfu mL^−1^) of strain XZL-8 was prepared as described above. Three treatments were set up as follows: CK1, 2 mL sterile water was uniformly spread on scalded area and its surrounding of the twigs, and only sterile PDA disks (6 mm) were placed on the scalded area of the twigs. CK2, 2 mL sterile water was uniformly spread on scalded area and its surrounding of the twigs, and fungal disks of *C. chrysosperma* (6 mm) were placed on the scalded area of the twigs. TR, 2 mL fermentation broth of strain XZL-8 was uniformly spread on scalded area and its surrounding of the twigs, and fungal disks of *C. chrysosperma* (6 mm) were placed on the scalded area of the twigs. All disks were covered by degreasing cotton wetted with sterile water and wrapped with preservative film. The twigs were placed vertically in beakers for hydroponics. Sterile absorbent cotton with sterile water was placed in the bottom of the beakers to provide adequate moisture and maintain a high relative humidity. The beakers were wrapped with preservative film and incubated at 25 ± 1 °C. At 2 days post-inoculation, the degreasing cotton and preservative film was removed. Each treatment consisted of 12 twigs with three replicates (four twigs per replicate). The development of disease symptoms and the formation of fruiting-bodies on the branches were observed at 14 d, 21 d, and 28 d after inoculation. The length and width of lesions were measured at 28 d after inoculation to calculate lesion area. The lesion area was calculated using the following formula:


Lesion Area(S)=π×Length×Width/4

The control effect was computed by the following equation:


$$\text{Control Effect}\;(\%)=100\times [\begin{array}{l} (S_{\mathrm{CK}2}–S_{\mathrm{CK}1})–\\ \left(S_{\mathrm{TR}}–S_{\mathrm{CK}1}\right) \end{array}]/(S_{\mathrm{CK}2}–S_{\mathrm{CK}1})$$

### 2.11 Statistical analysis

The mean values and standard deviations were calculated and statistically compared using analysis of variance (ANOVA) and Duncan’s multiple range tests (*p* ≤ 0.05) using SPSS software version 20.0 (SPSS Inc., Chicago, Illinois).

## 3 Results

### 3.1 Isolation and antagonistic effect evaluation of antagonistic strains

A total of 74 bacterial isolates were obtained from rhizosphere soil samples, were screened for antagonistic potential against *C. chrysosperma*. Only 12 were found to have an antagonistic potential against *C. chrysosperma*, and three strains (XZL-8, SY-15 and X2) showed strong inhibition on *C. chrysosperma*. Among these, strain XZL-8 showed the highest level of antifungal activity, as it inhibited *C. chrysosperma* at a distance of 18.00 mm (Table 2; Figure 1). Strain XZL-8 has been deposited in the China General Microbiological Culture Collection Center (no. 12555).

**Table 2:** Effect of rhizobacteria isolates against *C. chrysosperma in vitro*.

Isolates	Inhibition distance (mm)
DWZ-10	7.00 ± 0.70d
DWZ-8	6.50 ± 0.53de
X2	11.60 ± 0.89c
SY-13	5.60 ± 0.59ef
SY-15	13.20 ± 1.10b
SY-17	4.80 ± 0.84 fg
SY-8	3.84 ± 0.48 g
XZL-8	18.00 ± 0.71a
XZQ-1	6.00 ± 1.22de
XZQ-15	5.80 ± 0.45def
XZQ-23	6.40 ± 1.34de
XZQ-26	2.72 ± 0.80 h

Data are presented as mean ± SE. Different letters indicate significant differences between treatments (one-way ANOVA, *p* < 0.05).

**Figure 1:**
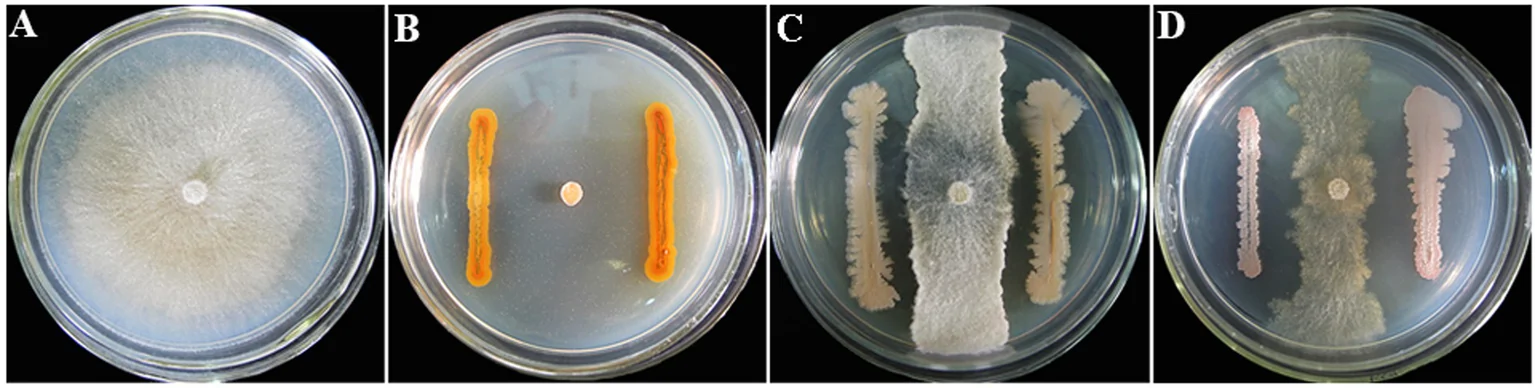
*In vitro* antagonistic effects of three strains against *C. chrysosperma* by dual-culture assay. **(A)** Mycelial growth of *C. chrysosperma* cultured on PDA plate. **(B–D)** Dual culture of *C. chrysosperma* with stains XZL-8, SY-15, X2, respectively.

### 3.2 Genomic characteristics and functional annotation of strain XZL-8

To elucidate the genetic basis underlying the biocontrol potential of strain XZL-8, whole-genome sequencing was performed, followed by comprehensive analyses of genomic features, functional gene annotation and putative antifungal secondary metabolites. Genome feature analysis and functional annotation (GO/COG/KEGG) and secondary metabolite gene cluster analysis (antiSMASH) were performed.

#### 3.2.1 Genome feature analysis

The complete genome of strain XZL-8 consisted of a single circular chromosome of 6, 967, 966 bp, with a G + C content of 62.87%. No ambiguous bases (N) were detected in the final assembly. Hybrid sequencing was performed, yielding Illumina short-read coverage of ~172-fold and Oxford Nanopore long-read coverage of ~269-fold. A total of 10 prophages, 69 tRNAs and 16 rRNAs (5S, 16S and 23S rRNA) were identified in this genome. A total of 6,305 protein-coding genes were predicted, and 6,113, 1,471, 3,569, 5,235 and 3,914 of them were assigned to the NR, GO, KEGG, eggNOG and Swiss-Prot databases, respectively (Figure 2; Table 3).

**Figure 2:**
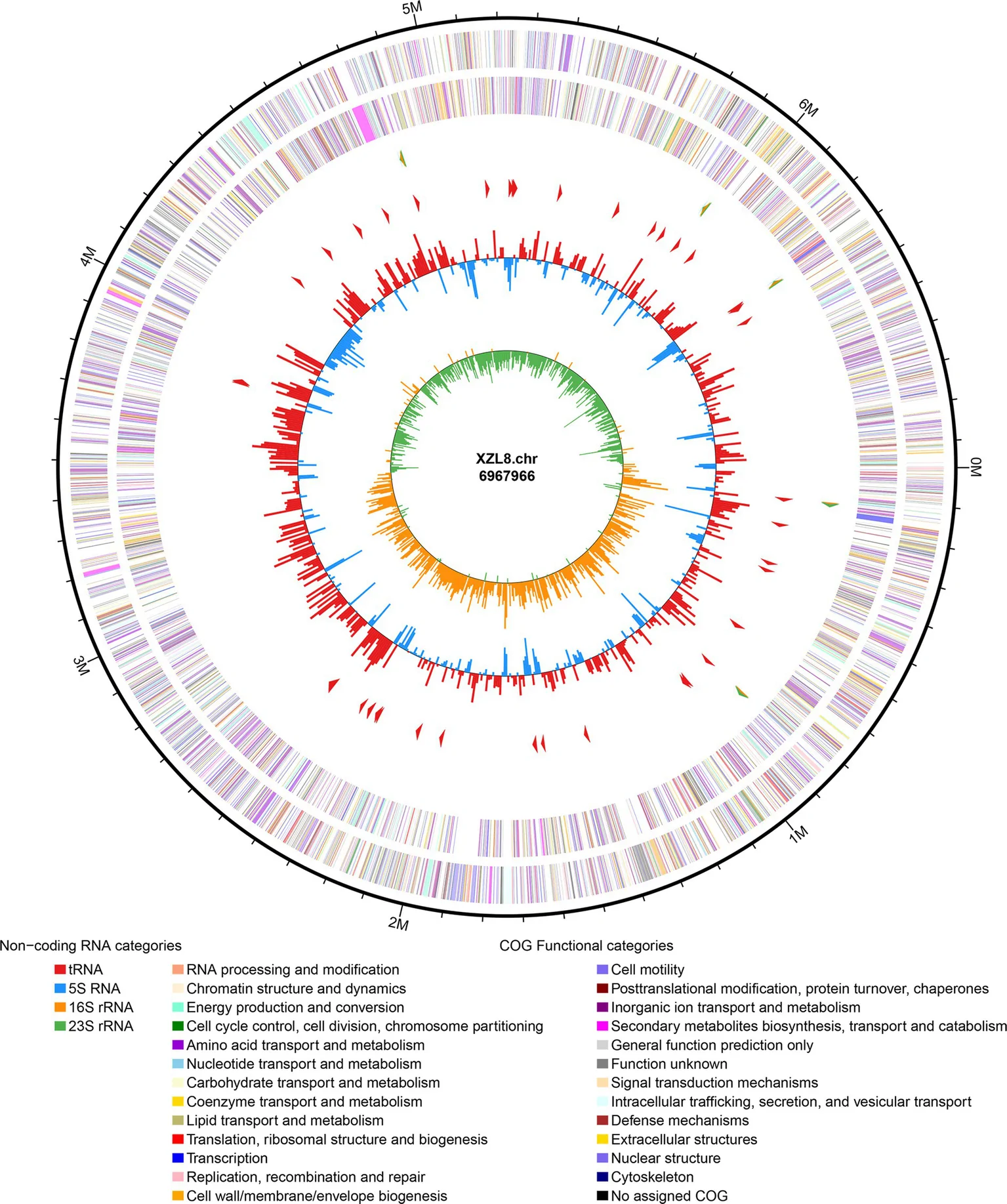
Circular genomic map of strain XZL-8. From the outside to the inside, the circles represent genome size, CDSs on the positive strand, CDSs on the negative strand, rRNAs and tRNAs, GC content, and GC skew, respectively.

**Table 3:** General genomic characteristics of strain XZL-8.

Feature	Value
Genome size (bp)	6,967,966
G + C content (%)	62.87
tRNA	69
5S rRNA	6
16S rRNA	5
23S rRNA	5
Total number of genes	6,305
Genes assigned to NR	6,113
Genes assigned to GO	1,471
Genes assigned to KEGG	3,569
Genes assigned to eggNOG	5,235
Genes assigned to Swiss-Prot	3,914

#### 3.2.2 COG/GO/KEGG functional annotation

COG functional classification was performed with eggnog-mapper v5.0 and DIAMOND based on the eggNOG database. The predicted proteins of strain XZL-8 were distributed across 21 COG categories. Transcription was the most highly represented category, with 613 genes (10.53%). The subsequent dominant categories included amino acid transport and metabolism (584 genes, 10.03%), inorganic ion transport and metabolism (461 genes, 7.92%), energy production and conversion (367 genes, 6.30%), and cell wall/membrane/envelope biogenesis (367 genes, 6.30%). In addition, 885 protein-coding genes (15.20%) had no clear functional annotations (Figure 3).

**Figure 3:**
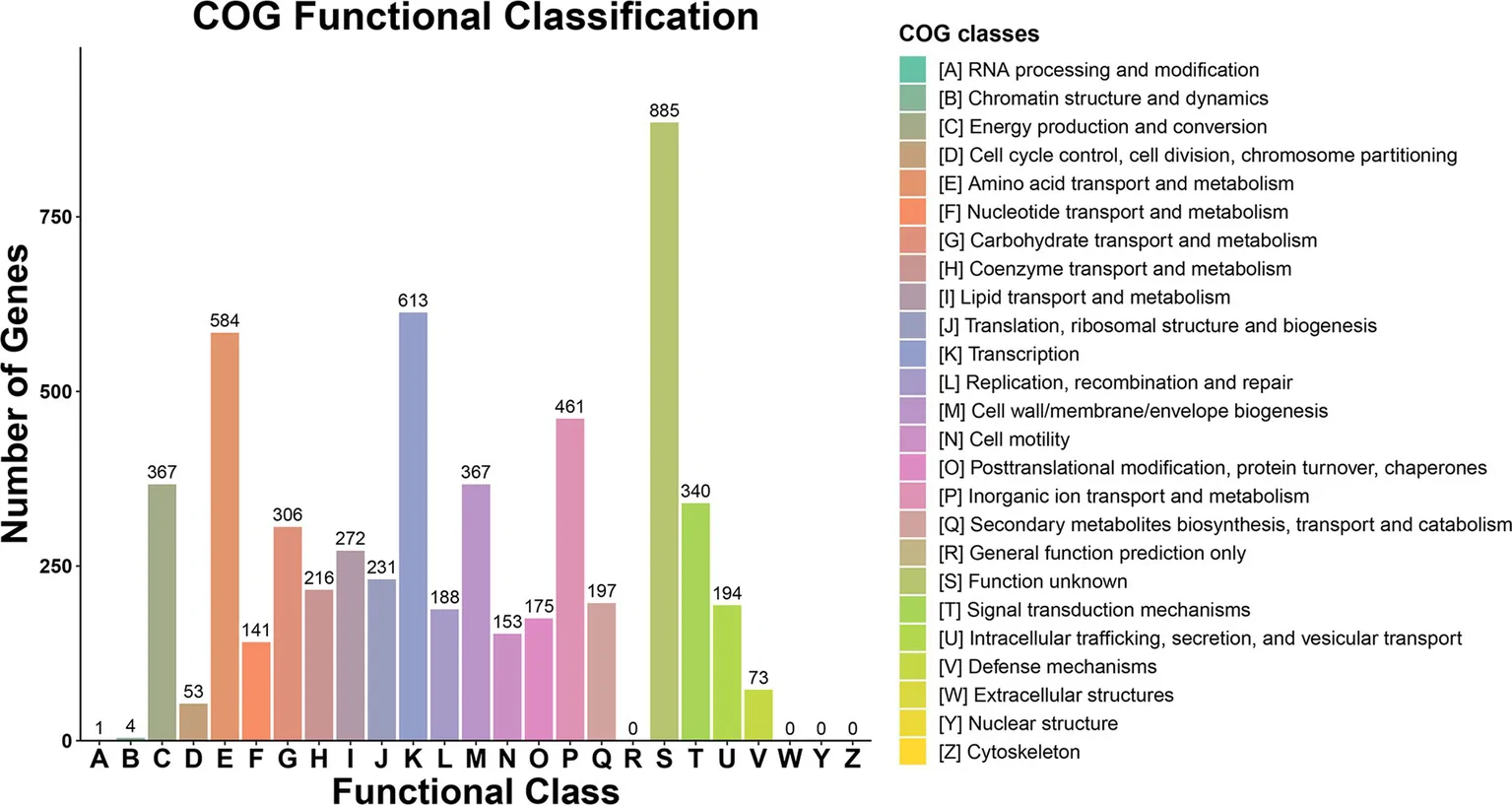
COG functional categories of strain XZL-8.

The genes of strain XZL-8 were annotated into three main GO categories: biological process (BP), cellular component (CC), and molecular function (MF). In the BP category, genes involved in metabolic process and cellular process were the most abundant. In the CC category, cellular anatomical entity and intracellular were the dominant terms. In the MF category, catalytic activity and binding were the most highly represented functions (Figure 4).

**Figure 4:**
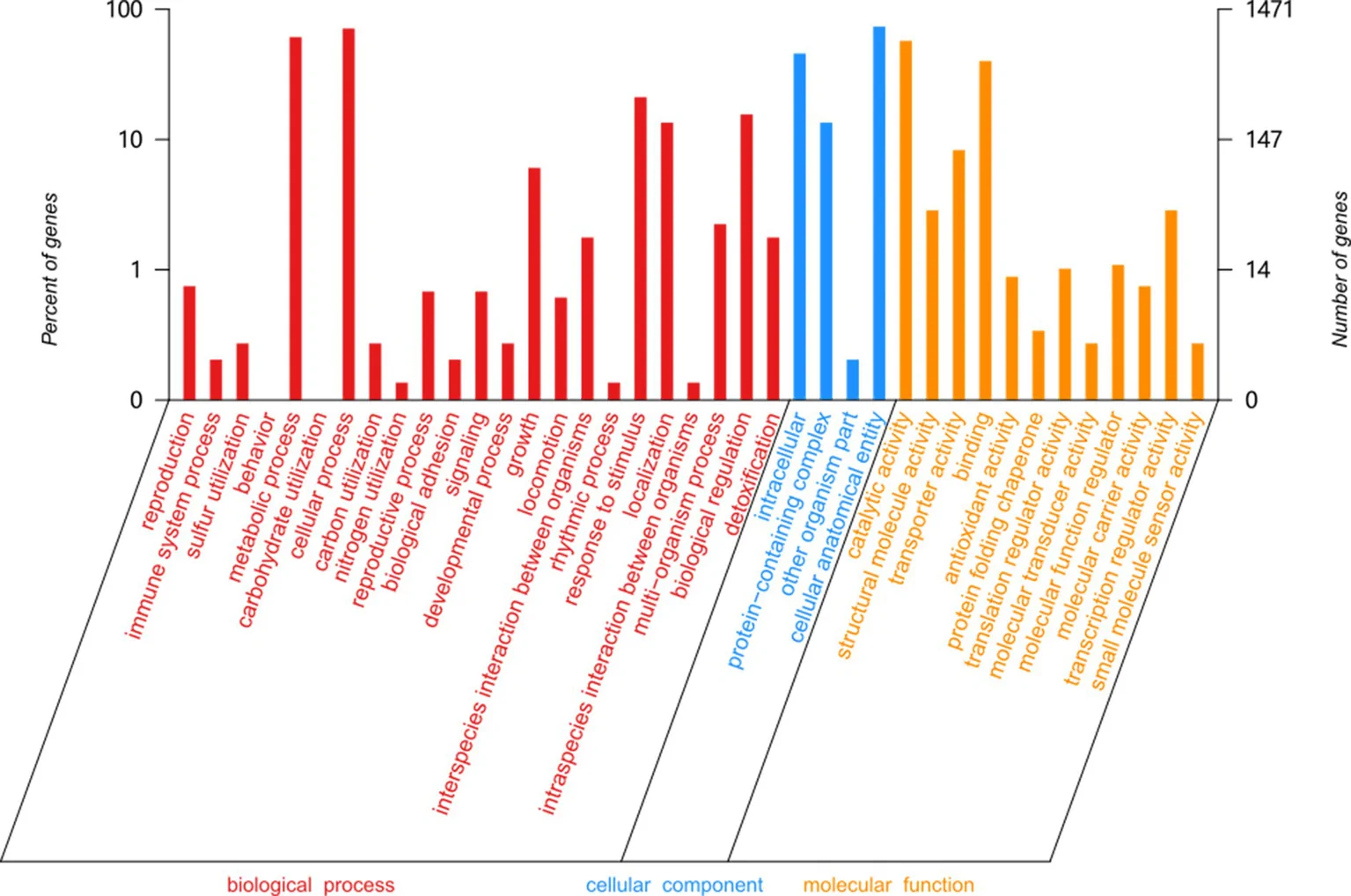
Gene Ontology categories of strain XZL-8.

The KEGG functional annotation of strain XZL-8 revealed that genes were predominantly enriched in the metabolism category (A), with Global and overview maps, amino acid metabolism, and carbohydrate metabolism being the most abundant subcategories. Genes related to environmental information processing (C), including membrane transport and signal transduction, and cellular processes (D), such as prokaryotic cellular community and cell motility, were also highly represented (Figure 5A). Within the top 20 pathways identified via enrichment analysis, metabolic pathways were the most significantly enriched. Ranking next were biosynthesis of secondary metabolites, microbial metabolism in diverse environments, and the two-component system (Figure 5B). These results collectively indicate that the strain possesses robust metabolic capabilities, strong environmental adaptability, and efficient signal transduction and material transport systems.

**Figure 5:**
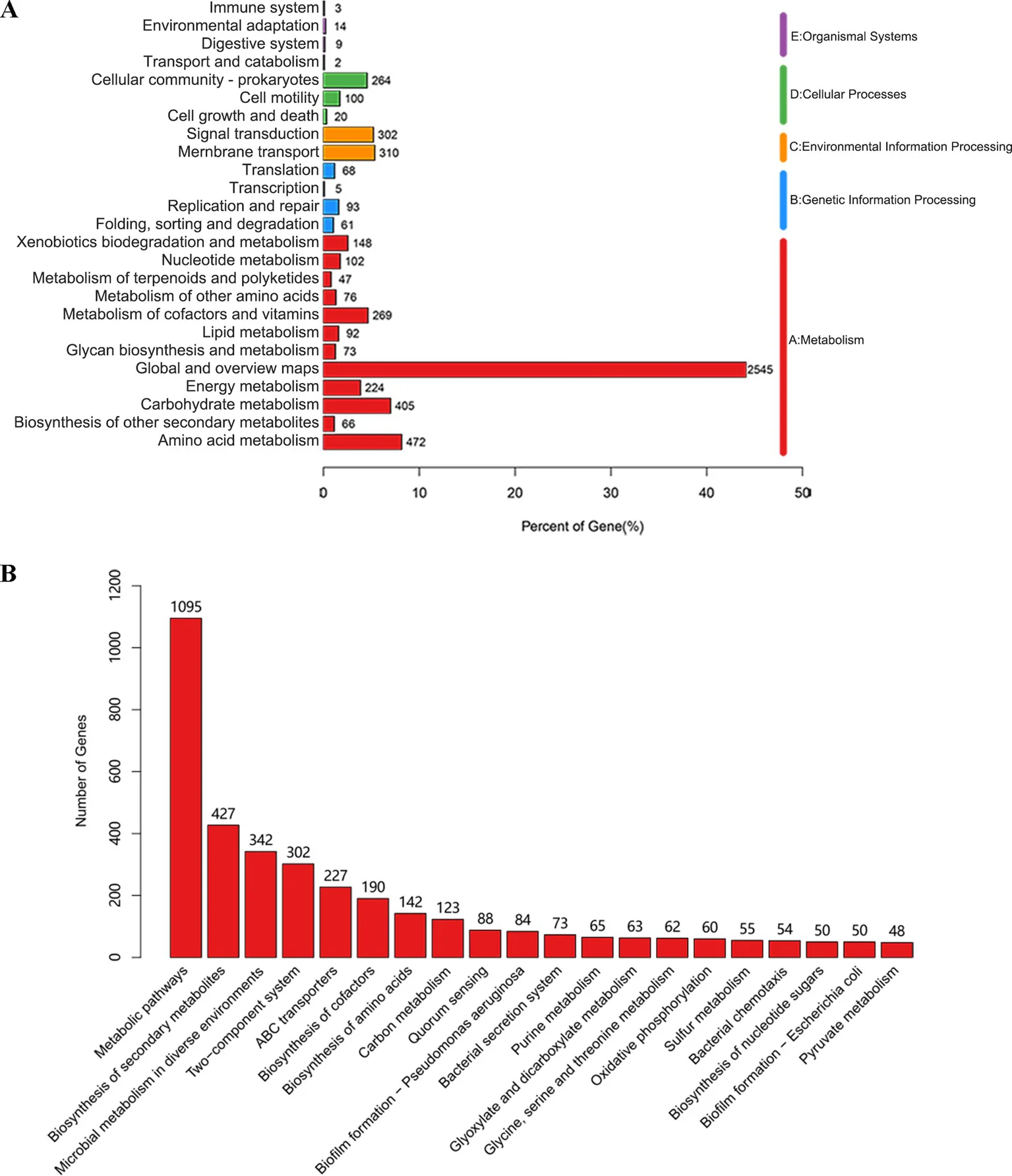
Functional annotation results of the strain XZL-8 genome based on the KEGG database. **(A)** Distribution of KEGG pathway categories. **(B)** Top 20 enriched KEGG pathway.

#### 3.2.3 Secondary metabolite gene clusters

The prediction of secondary metabolite biosynthetic gene clusters (BGCs) in the complete genome of strain XZL-8 was performed using the antiSMASH online server with relaxed strictness and all comparison modules activated (Table 4). A total of 22 candidate secondary metabolic regions were identified, covering diverse BGC classes including arylpolyene, hydrogen-cyanide, terpene, NI-siderophore, NRPS, hserlactone and phenazine. Among these, the hydrogen cyanide and pyrrolnitrin clusters obtained high similarity confidence, with similarity scores of 0.85 and 0.99, respectively.

**Table 4:** AntiSMASH-predicted secondary metabolite BGCs in strain XZL-8.

Type	From	To	Similarity confidence	Most similar known cluster
NRPS-like	115,321	158,752	−	−
arylpolyene	517,906	561,526	Low	APE Vf
RiPP-like	1,453,321	1,464,214	−	−
RiPP-like	2,529,113	2,541,314	−	−
hydrogen-cyanide	2,702,634	2,715,606	High	hydrogen cyanide
hserlactone	2,769,949	2,790,608	−	−
RiPP-like	2,919,151	2,929,939	−	−
NRPS	3,182,371	3,232,423	−	−
terpene	3,325,493	3,351,094	Low	sodorifen
NI-siderophore	3,515,369	3,552,290	Low	schizokinen
hydrogen-cyanide	3,571,896	3,584,743	−	−
other	3,913,061	3,954,143	High	pyrrolnitrin
butyrolactone	4,112,459	4,123,460	−	−
butyrolactone	4,238,970	4,249,776	−	−
betalactone	4,539,945	4,563,193	−	−
NRP-metallophore, NRPS, resorcinol, ranthipeptide	4,734,556	4,833,433	Low	pyoverdine
NRPS	4,875,059	4,928,075	Low	pyoverdine
NAGGN	5,068,157	5,082,834	−	−
hserlactone	5,771,083	5,791,763	−	−
hserlactone, phenazine	5,863,642	5,886,428	Low	endophenazine A/endophenazine B
terpene-precursor	6,204,400	6,225,287	−	−
redox-cofactor	6,358,213	6,380,366	−	−

### 3.3 ANI and GGDC in genomic distance estimation

Strain XZL-8 exhibited the highest ANI value (98.4%) with *P. chlororaphis* subsp. *aureofaciens* DSM 50083, which was higher than the pairwise ANI values obtained from comparisons with the other subspecies (Figure 6A). The dDDH value between XZL-8 and *P. chlororaphis* subsp. *aureofaciens* DSM 50083 was 89.0%. By contrast, the dDDH values between XZL-8 and *P. chlororaphis* subsp. *chlororaphis* DSM 50083, *P. chlororaphis* subsp. *aurantiaca* DSM 19603, *P. chlororaphis* subsp. *piscium* DSM 21509, and *P. chlororaphis* subsp. *phenazini* S1Bt23 were 59.9, 74.7, 63.2 and 60.4%, respectively (Figure 6B). Combined with the ANI and dDDH results, strain XZL-8 was identified as *P. chlororaphis* subsp. *aureofaciens*.

**Figure 6:**
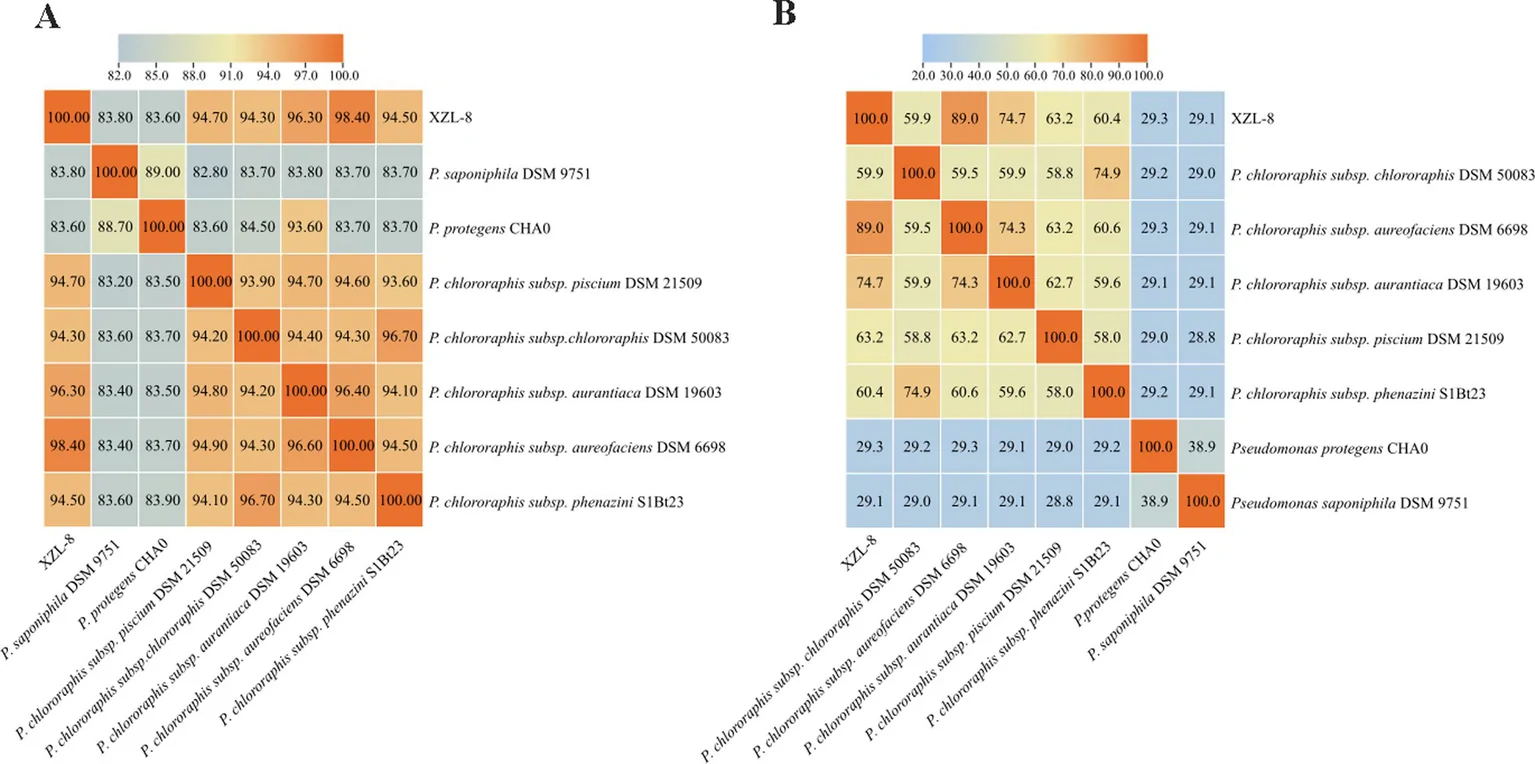
Heatmap analyses of ANI **(A)** and dDDH **(B)** values (%).

### 3.4 Morphological, physiological, biochemical, and molecular identification of strain XZL-8

Strain XZL-8 forms orange-red, opaque, smooth and circular colonies with central convexity and entire margins on King’s B (KMB) medium (Figure 7A). It is an asporogenous, flagellated and motile Gram-negative bacillus, with cell sizes of approximately 0.8–1.0 μm × 1.2–2.3 μm (Figure 7B). Physiological and biochemical characterization revealed that strain XZL-8 was able to grow at temperatures ranging from 4 °C to 37 °C, with an optimal growth temperature of 30 °C. Its pH growth range was 5.0–9.0, and the optimal pH for proliferation was 6.0–7.0. The strain could survive and propagate in NB medium supplemented with 0.2–3% NaCl. Biochemical assays yielded positive results for catalase, oxidase, nitrate reduction, gelatin liquefaction, and the utilization of glucose, D-xylose, mannitol and sodium citrate, whereas negative results were observed in tests of lecithinase, indole production, hydrogen sulfide generation, Voges-Proskauer, methyl red, phenylalanine deaminase, starch hydrolysis, and anaerobic growth in glucose agar (Table 5). Strain XZL-8 is a strictly aerobic bacterium that can decompose gelatin but lacks starch-hydrolyzing capacity, with a broad spectrum of available carbon sources.

**Figure 7:**
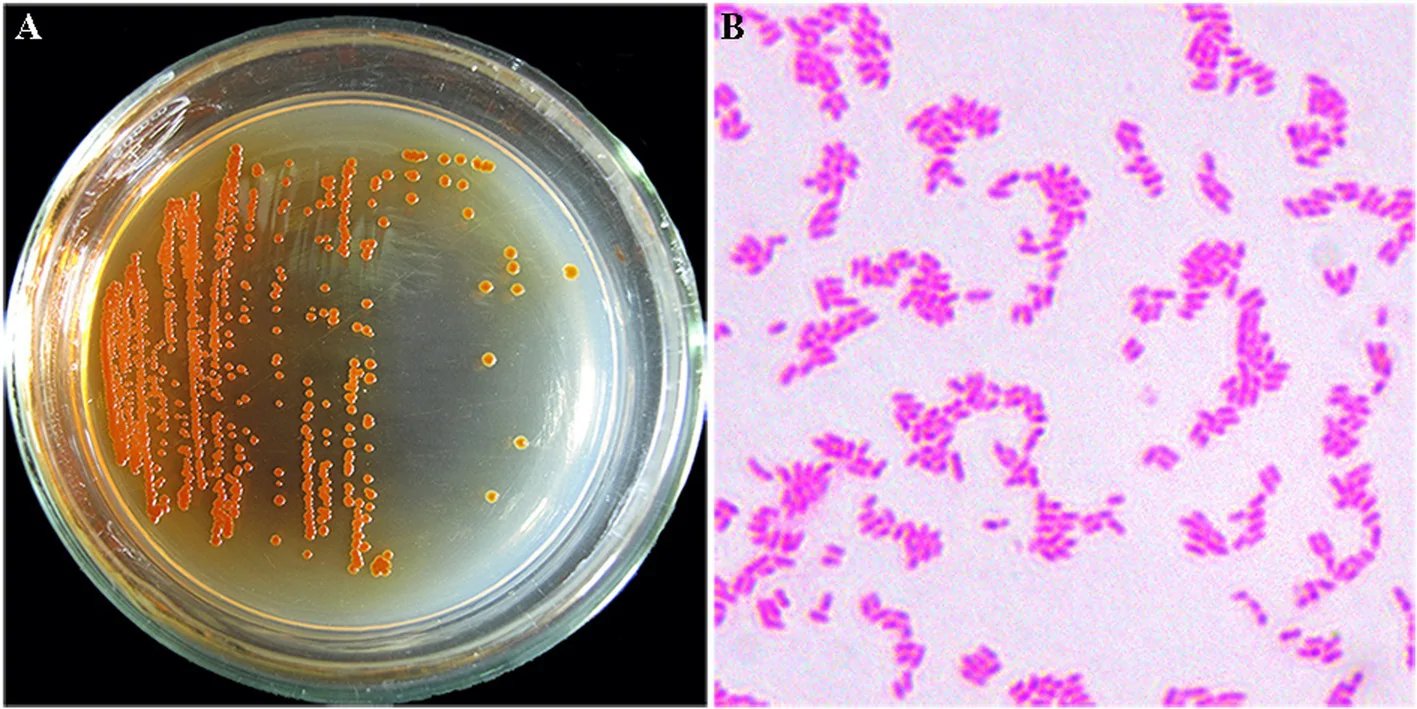
Colony morphological characteristics of strain XZL-8. **(A)** Colony morphology of bacterial strain XZL-8 on King’s B (KMB) medium. **(B)** Gram staining of XZL-8.

**Table 5:** Culture conditions and physiology-biochemical characteristics of strain XZL-8.

Indicator	Result
Gram staining	−
Mobility	+
Growth at 4 °C	+
Growth at 10 °C	+
Growth at 30 °C	+
Growth at 41 °C	−
Growth at pH 4	−
Growth at pH 5.7	+
Growth at pH 10	−
Growth with 0.2% (w/v) NaCl	+
Growth with 2% (w/v) NaCl	+
Growth with 5% (w/v) NaCl	−
Liquefication of gelatin	+
Hydrolysis of starch	−
Catalase test	+
Lecithinase test	−
Oxidase test	+
Reduction of nitrates	+
Production of indole	−
Production of hydrogen sulfide	−
Voges-Proskauer reaction	−
Utilization of glucose	+
Utilization of D-xylose	+
Utilization of Mannitol	+
Utilization of Sodium citrate	+
Anaerobic growth in glucose agar	−
Methyl red reaction	−
Phenylalanine deaminase test	−

+, positive, −, negative.

GBDP phylogenetic analysis based on the 16S rRNA gene indicated that the strain XZL-8 clustered within the *P. chlororaphis* clade and formed a subclade (bootstrap = 92) together with *P. chlororaphis* subsp. *aurantiaca* DSM 19603, *P. chlororaphis* subsp. *aureofaciens* DSM 6698 and *P. chlororaphis* subsp. *phenazini* S1Bt23 (Figure 8A). In contrast, the whole-genome-based GBDP phylogenetic tree demonstrated that this strain formed a distinct well-supported subclade with *P. chlororaphis* subsp. *aureofaciens* DSM 6698, supported by a bootstrap value of 86 (Figure 8B). The inconsistency in phylogenetic topologies derived from two datasets can be mainly attributed to the high sequence conservation of the 16S rRNA gene among different subspecies of *P. chlororaphis*, which leads to low discriminatory power for closely related subspecies. Meanwhile, frequent horizontal gene transfer and genetic recombination occur across subspecies of this species. Compared with the single 16S rRNA marker gene, phylogenomic trees constructed from genome-wide core genes provide a more accurate and reliable inference of the evolutionary relatedness and taxonomic assignment of bacterial strains. Integrating physiological, biochemical and phylogenomic evidence, strain XZL-8 was finally identified as *P. chlororaphis* subsp. *aureofaciens*.

**Figure 8:**
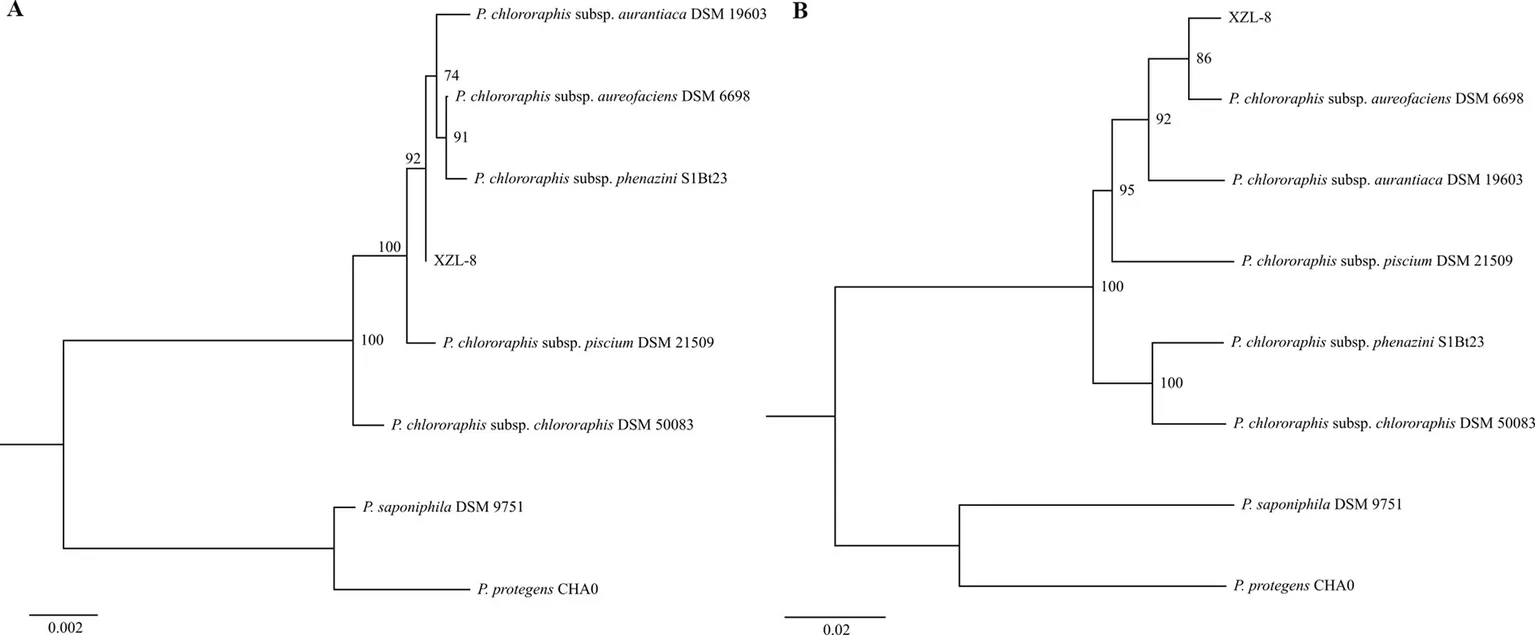
Phylogenetic analysis of strain XZL-8. **(A)** GBDP tree based on 16S rRNA gene sequences. **(B)** GBDP phylogenomic tree derived from whole-genome distances.

### 3.5 *In vitro* antifungal activity analysis of strain XZL-8

Fermentation broth and sterile culture filtrate of strain XZL-8 collected at different fermentation times were evaluated for effects on hyphal growth of *C. chrysosperma*. The result showed that fermentation broth and sterile culture filtrate of strain XZL-8 could inhibit the hyphal growth of *C. chrysosperma* (Figures 9A–C). Fermentation time (1–7 d) had no impact on the inhibition rate of fermentation broth, which completely inhibited *C. chrysosperma* mycelial growth. For sterile culture filtrates, inhibition rate varied noticeably over days 1–5, peaking at 93.55% on day 5. Inhibition rates remained stable at approximately 93% with no significant differences from days 5 to 7 (Figure 10A). Strain XZL-8 produced volatiles that reduced the hyphal growth of *C. chrysosperma*, with inhibition rate of 81.62% at 48 h (Figures 9D,E). Moreover, when the colony diameters of *C. chrysosperma* were measured every 12 h, the hyphal growth was significantly inhibited after 24 h by the volatiles produced by strain XZL-8, and there was a gradual decrease in the inhibition rate from 12 to 48 h (Figure 10B).

**Figure 9:**
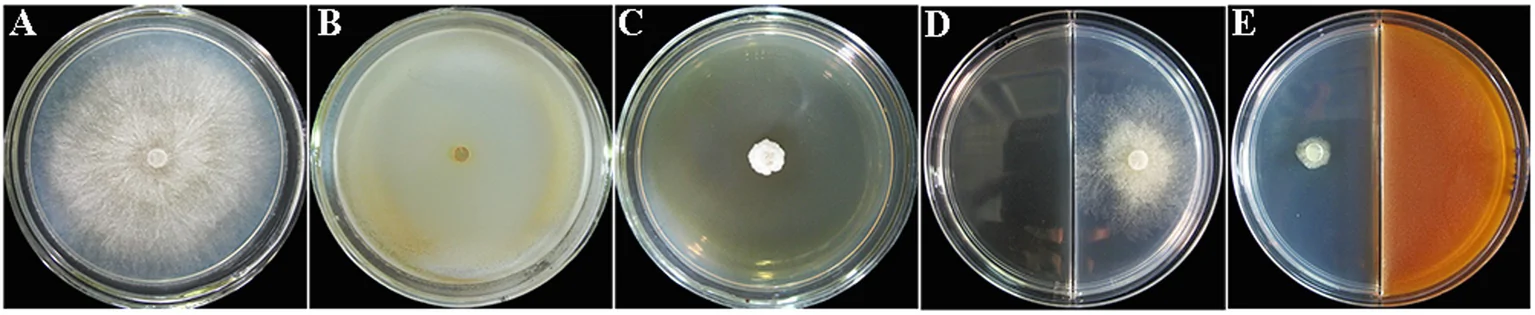
Antifungal activity analysis of strain XZL-8 against *C. chrysosperma*. **(A)** *C. chrysosperma* mycelial growth cultured on PDA. **(B)** Antifungal activity of strain XZL-8 fermentation broth. **(C)** Antifungal activity of strain XZL-8 sterile culture filtrate. **(D)** Volatile control: left, KMB plate, right, *C. chrysosperma* inoculated PDA. **(E)** Volatiles antifungal activity: left, pathogen-inoculated PDA, right, KMB plate with strain XZL-8 broth.

**Figure 10:**
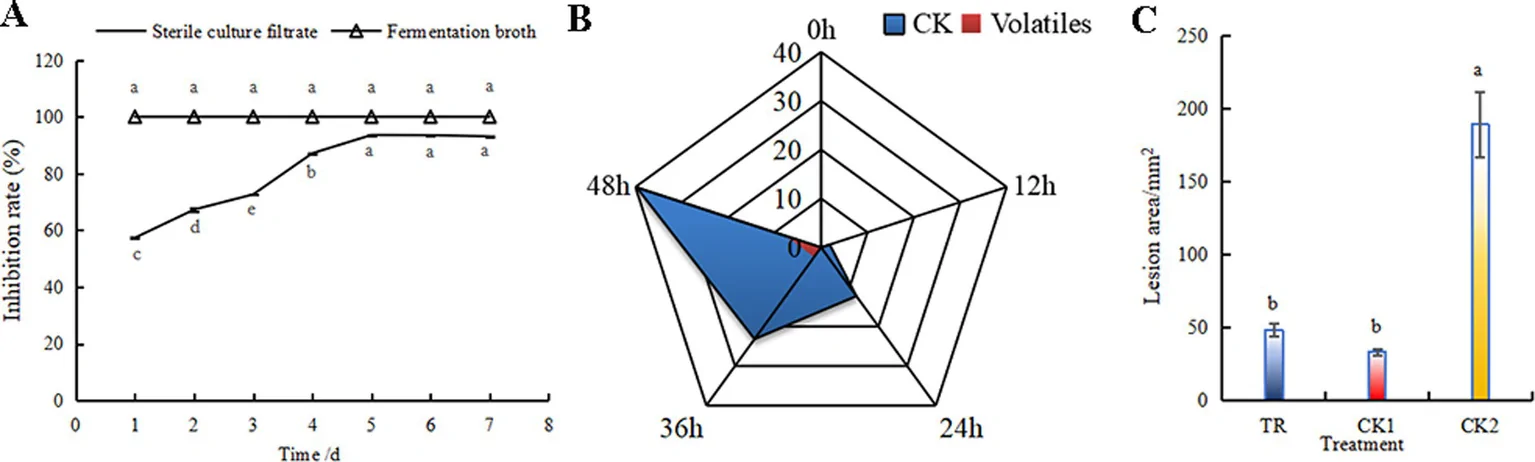
Antifungal performance of strain XZL-8 against *C. chrysosperma*. **(A)** Antifungal effects of 1–7 d fermentation broth and sterile culture filtrate. **(B)** Radar plot of 0–48 h mycelial growth; red, volatiles, blue, CK. **(C)** Lesion area of *C. chrysosperma* under different treatments. Different letters represent significant differences (*p* < 0.05), while the same letters indicate no significant differences.

### 3.6 Hydrogen cyanide (HCN), hydrolytic enzyme and phosphate solubilization production

Hydrogen cyanide (HCN) and hydrolytic-enzyme production by strain XZL-8 were evaluated using multiple biochemical assays. A distinct colour change of the indicator filter paper from yellow to light-brown indicated moderate HCN production by strain XZL-8 (Figures 11A,B). After 7 days of incubation, faint milky halos formed around XZL-8 colonies on Tween-80 agar, revealing esterase secretion. Clear degradation zones were also observed on skim-milk agar, demonstrating protease production (Figures 11C,D). These phenotypic assays confirmed that strain XZL-8 is capable of secreting extracellular esterase and protease. Further plate-based assays revealed that strain XZL-8 solubilized insoluble inorganic phosphate on NBRIP agar plates, producing prominent transparent halos surrounding its colonies (Figure 11E).

**Figure 11:**
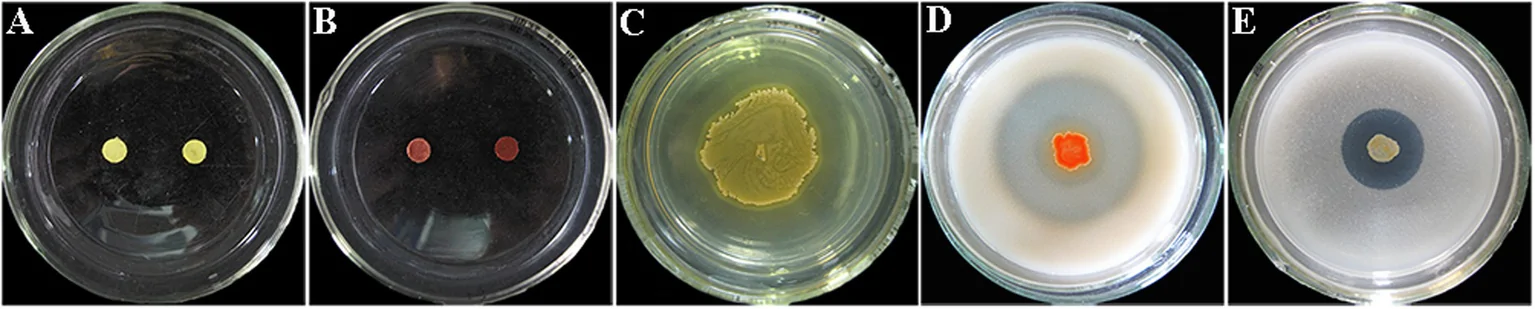
Assay of functional metabolites. **(A)** Negative control without hydrogen cyanide (HCN) production. **(B)** HCN production. **(C)** Esterase activity assay on Tween 80 agar. **(D)** Protease activity assay on skim milk agar. **(E)** Phosphate-solubilizing assay on NBRIP medium. **(F)** Indole-3-acetic acid (IAA) production assay.

### 3.7 Biocontrol efficacy of strain XZL-8 on detached twigs

Fermentation broth of strain XZL-8 was applied on poplar twigs to determine its effect on disease development caused by *C. chrysosperma*. The twigs treated by sterile water and fungal disks of *C. chrysosperma* (CK2) appeared obviously sunken lesion and formed conidiomata after 14 days of inoculation. The color of the lesion on the twigs changed from the original green to brown. After 21 days and 28 days of inoculation, the lesion continue to expanded on the twigs, and formed more and more conidiomata. Many conidiomata gradually overflowed with orange cirrus after 28 days of inoculation (Figure 12). However, After 14 days, 21 days and 28 days of inoculation, no obvious lesions were found in twigs of CK1 (Treated by sterile water and sterile PDA disks) and TR (Treated by fermentation broth and fungal disks of *C. chrysosperma*). At 28 days post-inoculation, no significant difference in lesion area was observed between CK1 and TR, whereas both treatments exhibited a significant difference compared with CK2 (*p* < 0.05) (Figure 10C). The biocontrol efficacy reached 90.3%. These results showed that fermentation broth of XZL-8 could effectively inhibit the development of *C. chrysosperma* on detached twigs of poplar.

**Figure 12:**
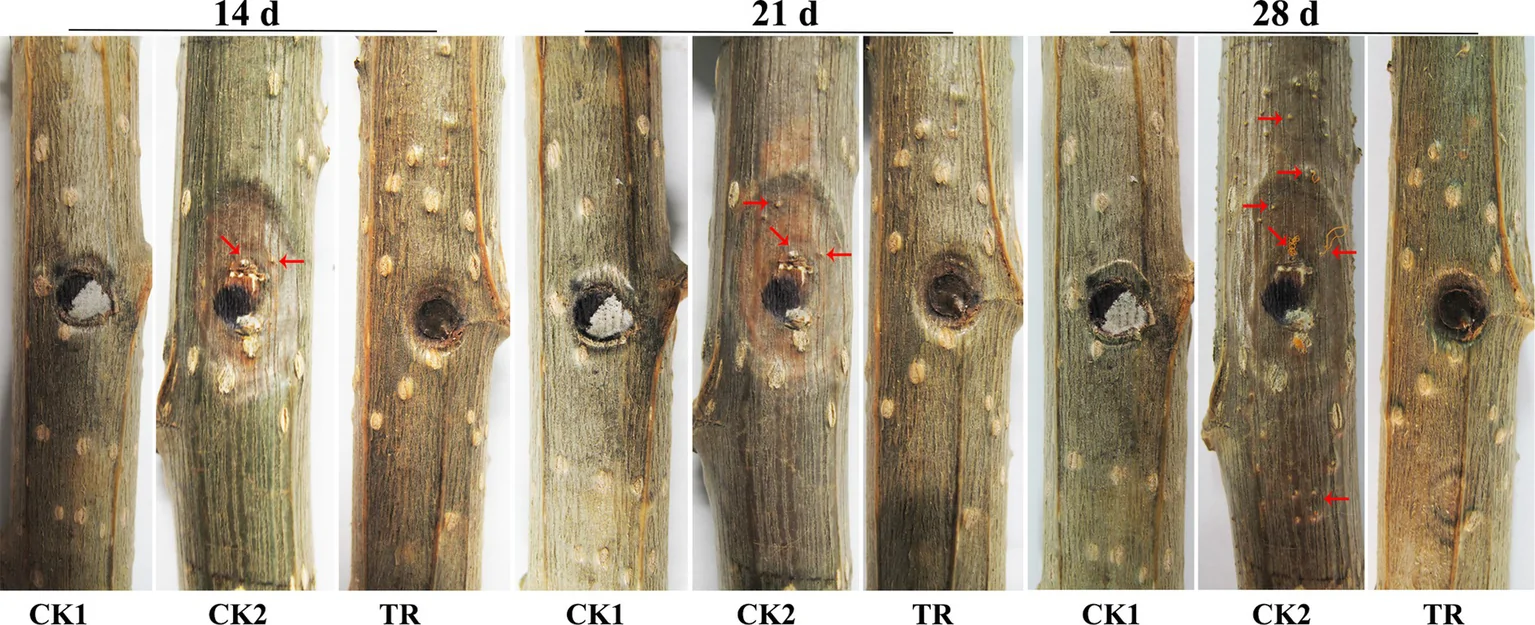
Biocontrol efficacy of strain XZL-8 fermentation broth against poplar canker caused by *C. chrysosperma* on detached poplar twigs. CK1, Treated by sterile water and sterile PDA disks; CK2, Treated by sterile water and fungal disks of *C. chrysosperma*; TR, Treated by fermentation broth and fungal disks of *C. chrysosperma*. Red arrows: conidiomata.

## 4 Discussion

In this study, the tested biocontrol strain XZL-8 was identified as *P. chlororaphis* subsp. *aureofaciens* using polyphasic taxonomy and whole-genome sequence analysis. *P. chlororaphis* subsp. *aureofaciens* represents a well-studied plant-beneficial rhizobacterium with dual capacities for pathogen biocontrol and plant growth promotion. Widely distributed in natural environments, this subspecies has attracted extensive attention owing to its ability to synthesize phenazine antibiotics, siderophores, indole-3-acetic acid (IAA), and multiple extracellular hydrolytic enzymes (Gross and Loper, 2009; Du et al., 2026).

Genome-wide mining predicted a total of 22 putative biosynthetic gene clusters (BGCs) for secondary metabolites in *P. chlororaphis* subsp. aureofaciens XZL-8, responsible for the biosynthesis of bioactive compounds including pyrrolnitrin, hydrogen cyanide, phenazines and siderophores. Pyrrolnitrin interferes with mitochondrial electron transport and disrupts membrane integrity in phytopathogens, inhibiting fungal mycelial elongation and spore germination (Huang et al., 2018). *In vitro* picric acid colorimetric assays confirmed that strain XZL-8 is capable of hydrogen cyanide (HCN) production, which exerts broad-spectrum antagonistic effects by inhibiting key respiratory enzymes and blocking the aerobic energy metabolism of phytopathogens (Anand et al., 2020). Genomic analysis revealed that strain XZL-8 harbors the intact core HCN biosynthetic operon hcnA-hcnB-hcnC, whereas the auxiliary gene hcnD is absent (Supplementary Table 1). In pseudomonads, the three structural genes hcnABC encode the subunits of HCN synthase and are sufficient to support cyanide biosynthesis; hcnD only participates in accessory regulatory functions and is dispensable for HCN formation (Laville et al., 1998). Consistent with this genomic feature, the positive yellow-to-brown color shift of picric acid filter paper further validated the strain’s cyanogenic capacity, indicating its promising biocontrol potential via HCN-mediated inhibition of plant pathogens. Phenazines serve as signature antagonistic metabolites of this subspecies and can significantly suppress the proliferation of diverse phytopathogens (Raio et al., 2011). The siderophore system enables efficient chelation of free ferric ions in the rhizosphere; iron competition restricts pathogen access to essential trace elements and hinders pathogen colonization and expansion on plant surfaces (Shao et al., 2024). Notably, 14 predicted BGCs share low sequence similarity with characterized clusters, and their corresponding metabolites remain unknown, suggesting that strain XZL-8 have abundant unexploited novel bioactive secondary metabolites.

Combining *in vitro* phenotypic assays and genomic sequencing, this study provides genomic clues for multiple plant-beneficial properties of strain XZL-8, revealing partial consistency between its genetic repertoire and observed functional phenotypes. All functional genes discussed in this section are listed in Supplementary Table 1. The acdS gene, encoding ACC deaminase, was detected, implying the genetic potential to degrade the ethylene-precursor ACC and thereby mitigate ethylene-mediated plant-growth inhibition and enhance plant stress adaptability (Saleem et al., 2007). Although the strain contains the inorganic phosphorus-solubilizing gene gcd, the absence of pqqA and pqqF disrupts complete PQQ cofactor biosynthesis, disabling the glucose oxidation-dependent phosphate solubilization pathway; the distinct transparent halo on NBRIP plates is presumably associated with secretion of organic acids such as citric and malic acid (An and Moe, 2016). In addition, abundant lipolytic genes (apeE, estA, lip-1, lipA, lipB) and proteolytic genes (npr, degS, degP) together with complete protein secretion systems in the genome, suggest a potential capacity for rhizospheric macromolecule degradation. This genomic feature is partially supported by the hydrolytic phenotypes observed on Tween 80 and skim-milk media, which may improve rhizosphere nutrient availability (Yu et al., 2023; Rosenau and Jaeger, 2000). Collectively, genome-based analysis reveals that XZL-8 harbors diverse genetic determinants associated with ethylene stress mitigation, nutrient mobilization and macromolecular degradation. While the strain displays obvious antagonistic activity against pathogens in laboratory assays, these plant-beneficial traits remain largely putative at present and require further plant-based bioassays for validation. XZL-8 represents a promising candidate for future sustainable plant-disease-control applications.

*In vitro* branch assays verified that the strain could stably colonize branch surfaces and markedly inhibit pathogen-induced lesion expansion. It exhibits prominent protective effects against woody plant branch diseases and is suitable for the biological control of infection-prone sites including pruning wounds and mechanical injuries. However, the *in vitro* system fails to simulate the complete physiological metabolism and induced disease resistance pathways of intact plants. This research only confirms the direct antibacterial function of the strain, and whether it can boost plant disease resistance through induced systemic resistance (ISR) requires further investigation.

Taken together, *P. chlororaphis* subsp. *aureofaciens* strain tested in this study possesses prominent disease-suppressive activity its whole-genome sequence provides valuable genetic information for dissecting the biocontrol mechanisms, metabolic pathways and environmental adaptability of this subspecies. *In vitro* branch assays further validated the application potential of this strain in the control of woody plant branch diseases. Future in-depth research can focus on the colonization dynamics of the strain, transcriptional regulation of key functional genes, optimization of microbial agent formulations, and field biocontrol efficacy evaluation, so as to facilitate its practical application in green agriculture and the prevention of forest and fruit tree diseases. Collectively, the findings of this study expand the reservoir of functional strains of *P. chlororaphis* subsp. *aureofaciens* and supply an outstanding candidate strain for the development of biocontrol microbial inoculants.

## 5 Conclusion

In this study, the biocontrol strain XZL-8 was identified as *P. chlororaphis* subsp. *aureofaciens* based on polyphasic taxonomy and whole-genome analysis. Genomic BGC prediction using antiSMASH identified 22 putative secondary metabolite biosynthetic gene clusters belonging to various types. Among them, the biosynthetic gene clusters for hydrogen cyanide and pyrrolnitrin showed high similarity scores of 0.85 and 0.99, respectively, suggesting that the strain harbors the genetic potential to produce antimicrobial compounds. Functional verification demonstrated that the strain inhibits *C. chrysosperma* pathogen growth through multiple strategies, including direct confrontation, extracellular active substance secretion, fermentative metabolites, and volatile compounds. Genomic mining revealed a suite of candidate genes associated with biocontrol and plant-beneficial traits, providing molecular clues for its biological activities. *In vitro* branch assays further confirmed its biocontrol potential against the pathogen. Collectively, strain XZL-8, which carries abundant antimicrobial biosynthetic gene clusters and exhibits multiple antagonistic phenotypes *in vitro*, represents a promising candidate resource for green forest disease management and microbial agent development.

## Data Availability

The data presented in this study are deposited in the NCBI GenBank database under accession number JCCMNM000000000 (https://www.ncbi.nlm.nih.gov/genbank/).

## Footnotes

1
http://www.usadellab.org/cms/uploads/supplementary/Trimmomatic

2
http://circos.ca/

3
http://uniprot.org

4
http://www.genome.jp/kegg/

5
http://www.ncbi.nlm.nih.gov/COG

6
https://antismash.secondarymetabolites.org/

7
http://lowelab.ucsc.edu/tRNAscan-SE

8
http://www.cbs.dtu.dk/services/RNAmmer/

9
https://www.ncbi.nlm.nih.gov/genome

10
http://jspecies.ribohost.com/jspeciesws/

11
https://tygs.dsmz.de
