# Supplementary material for: Whole-genome sequencing and biocontrol functional analysis of Pseudomonas chlororaphis XZL-8 for controlling poplar canker pathogen Cytospora chrysosperma
Source: Front Microbiol. 2026 Oct 2;17:1973011. doi: 10.3389/fmicb.2026.1973011 (PMC13681834; doi:10.3389/fmicb.2026.1973011)
Supplement: Supplementary file 1 [file Table_1.DOCX]

Supplementary Table 1 Genes associated with antifungal and plant growth-promoting activities in strain XZL-8

| XZL-8 antifungal and PGP activities description | Gene name | Gene annotation | Gene ID |
| --- | --- | --- | --- |
| HCN production | hcnA | Hydrogen cyanide synthase HcnA | XZL8002448 |
|  | hcnB | Hydrogen cyanide synthase HcnB | XZL8002449 |
|  | hcnC | Hydrogen cyanide synthase HcnC | XZL8002450 |
| Hydrolytic enzyme production | pepP | Xaa-Pro aminopeptidase | XZL8004324 |
|  | apeE, estA, lip-1 | outer membrane lipase/esterase | XZL8003515 |
|  |  | Phospholipase/lecithinase/hemolysin | XZL8003677 |
|  |  | Phospholipase/lecithinase/hemolysin | XZL8005712 |
|  | NAPEPLD | L-ascorbate metabolism protein UlaG, beta-lactamase superfamily | XZL8004722 |
|  | ptsO, npr | HPr family phosphocarrier protein | XZL8000933 |
|  | prtC | Hemolysin-type calcium-binding repeat-containing protein | XZL8002118 |
|  |  | serralysin | XZL8002354 |
|  |  | epralysin. Metallo peptidase. MEROPS family M10B | XZL8003268 |
|  | prtC, trhP | putative protease | XZL8004126 |
|  | hhoB, degS | Outer membrane stress sensor protease DegS | XZL8000953 |
|  | degP, htrA | serine peptidase | XZL8001466 |
|  | CARP, pepA | leucyl aminopeptidase | XZL8001075 |
|  |  | leucyl aminopeptidase | XZL8001075 |
|  | ampS, pepS, ampT | Leucyl aminopeptidase (aminopeptidase T) | XZL8001627 |
|  | pepN | aminopeptidase N | XZL8002716 |
|  | DPEP | peptidase M19 | XZL8004263 |
|  |  | peptidase M19 | XZL8005816 |
|  | pepP | Xaa-Pro aminopeptidase | XZL8004324 |
|  |  | aminopeptidase P Metallo peptidase. MEROPS family M24B | XZL8006022 |
| Phosphate metabolism | gcd | quinoprotein glucose dehydrogenase | XZL8005035 |
|  | pqqB | pyrroloquinoline quinone biosynthesis protein B | XZL8005752 |
|  | pqqC | pyrroloquinoline-quinone synthase PqqC | XZL8005753 |
|  | pqqD | Coenzyme PQQ synthesis protein D | XZL8005754 |
|  | pqqE | Coenzyme PQQ synthesis protein E | XZL8005755 |
| ACC deaminase | acdS | 1-aminocyclopropane-1-carboxylate deaminase | XZL8004095 |
